# Combined cardiac anomalies in Noonan syndrome: A case report

**DOI:** 10.1016/j.ijscr.2020.05.048

**Published:** 2020-05-30

**Authors:** Natraj Setty H.S., Shankar S., Rahul Patil, Santosh Jadhav, Yeriswamy M.C., Babu Reddy, Jayashree Kharge, T.R. Raghu, Sandeep Shankar, Sathwik Raj, Chethan N., Nithin M., C.N. Manjunath

**Affiliations:** Sri Jayadeva Institute of Cardiovascular Sciences and Research, Bangalore, Karnataka, India

**Keywords:** Noonan syndrome, 2D-Echocardiography, ASD, Biventricular hypertrophy, Case report

## Abstract

**Introduction:**

Noonan syndrome is the second most common syndromic cause of congenital heart disease. Most patients have an autosomal dominant inheritance, but some cases may be sporadic. Pulmonary stenosis is the most common cardiac manifestation in Noonan syndrome, associated with the atrial septal defect and hypertrophic cardiomyopathy. A combination of these three is present only in 5% of patients.

**Presentation of case:**

We report a case of a 21-year-old female who presented to our hospital concomitant cardiac lesions associated with pulmonary stenosis, atrial septal defect, and hypertrophic cardiomyopathy. This combination of cardiac defects is an infrequent manifestation of Noonan syndrome. The patient presented with complaints of exertion syncope over the past two years. 2D-Echocardiography showed biventricular hypertrophy, dysplastic pulmonary valve, severe pulmonary stenosis, asymmetric septal hypertrophy and large atrial septal defect. The genetic analysis report showed autosomal dominant inheritance with Ras/MAPK (mitogen-activated protein kinase) Positive.

**Discussion:**

Due to the wide spectrum of symptoms and presentations in Noonan cases, accurate clinical and genetic diagnosis, and comprehensive management of the disorder are strongly recommended.

**Conclusion:**

We have described a case of rare combination of cardiovascular defects in Noonan Syndrome with a view to achieve better insight into the disease course and advantages of timely treatment and follow up. Our patient is currently in follow-up after treatment with percutaneous balloon pulmonary valvuloplasty, has improved symptoms, and is awaiting heart transplant.

## Highlights

• Noonan syndrome is the second most common syndromic cause of CHD.
• We present a rare case of Noonan syndrome having a combination of ASD, PS and HCM.
• Patient underwent balloon valvotomy for pulmonary stenosis.
• The patient is under regular follow-up and awaiting heart transplant.

## 1 Introduction

Noonan syndrome was first recognized as a unique entity in 1963 when Jacquline Anne Noonan described a series of patients with pulmonary stenosis and characteristic physical appearance with short stature, webbed neck, wide-spaced eyes, and low-set ears. Noonan Syndrome occurs in 1:1000–1:2500 live births. Popularly termed as the male version of Turner’s syndrome, it equally affects girls and boys, but the latter present early with cryptorchidism. It is genetically heterogeneous with at least eight causative genes acting in the RAS/MAPK signaling pathway [1]. It is clinically heterogeneous and can present at any age. There is a characteristic facial appearance (broad forehead, ptosis, low set ears, widely spaced eyes, low posterior hairline, webbed neck, pectus excavatum, bleeding diathesis, lymphatic issues, learning disability and cryptorchidism [2]. More than 80 percent of patients have cardiac involvement, most often pulmonary valve stenosis (PS) with dysplastic pulmonary valve. They have a higher re-intervention rate of approximately 65 % after percutaneous balloon pulmonary valvuloplasty (PBPV). Atrial septal defects (ASD) are also frequent, and 20% of patients have hypertrophic cardiomyopathy (HCM). Other less common manifestations are ventricular septal defect (VSD), Patent ductus arterioles (PDA), Mitral valve prolapse (MVP), Aortic root dilation, Coarctation of aorta (COA), tetralogy of fallot (TOF), persistence of left superior vena cava to the coronary sinus, complete atrioventricular canal defects, congenital MS (mitral stenosis), Ostiumprimum ASD, double aortic arch, anomalous pulmonary venous return, sinus venous defect, bilateral superior vena cavae, and quadricuspid aortic valve.

A combination of PS, ASD, and HCM is present only in 5% of patients. Recently, a patient with Noonan syndrome presented with this rare combination of congenital heart defects, this is therefore a unique case, warranting follow-up after treatment to help gain further insight in the disease course. This case has been reported in line with the SCARE criteria [3].

## 2 Presentation of case

A twenty-one year old female presented with complaints of six episodes of exertional syncope over the past two years. She denied having chest pain, palpitation, paroxysmal nocturnal dyspnoea, or swelling of feet. There was no history of polyarthralgia and fever during childhood. She was born from a non-consanguineous marriage, and there was no history of exposure to teratogens during the antenatal period. She had typical developmental milestones, except for poor performance at school. No significant family history was present. On examination, she had short stature (150 cm), low set ears, a broad forehead, kyphoscoliosis, and broad chest. Pulse, blood pressure, and jugular venous pressure were normal. There was no pallor, cyanosis, clubbing, or pedal edema. Double apical impulse was felt in the left fourth intercostal space in the midclavicular line, heaving in character. Grade II left parasternal heave was present. A systolic thrill was palpable in the left upper sternal border. On auscultation, the first heart sound was normal, but the second heart sound heard was muffled. Fourth heart sound was heard. A loud crescendo-decrescendo murmur (Grade VI) was present at the apex, which varied With dynamic auscultation, suggesting HCM. Routine blood investigations were normal. The electrocardiogram showed biventricular hypertrophy. HOLTER monitoring showed no episodes of ventricular tachycardia, however few runs of sinus tachycardia were noted.

**Fig. 1:**
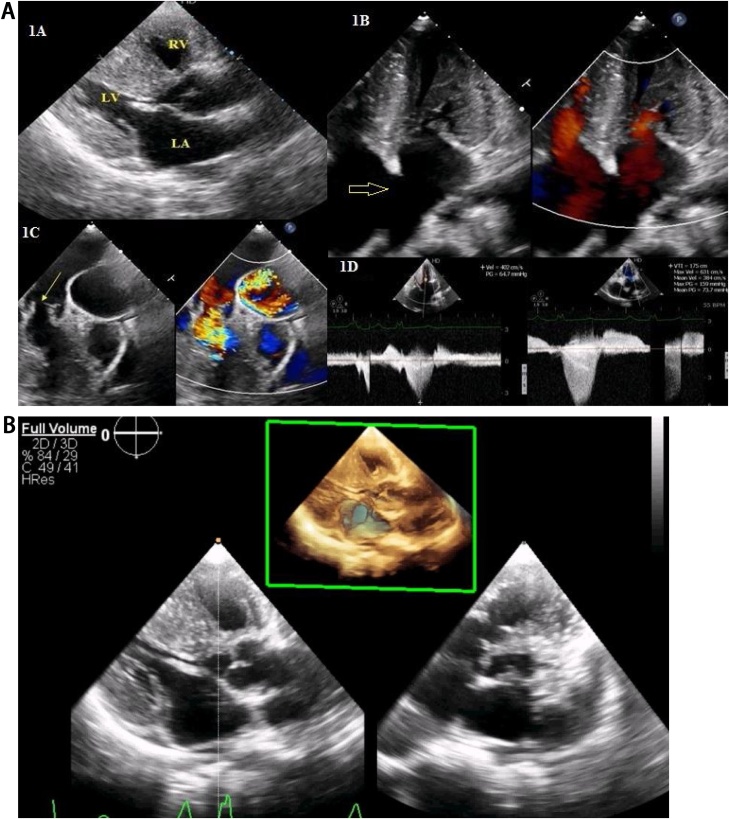
A: 2D-Echocardiography parasternal long axis showing biventricular hypertrophy. B: 2D-Echocardiography apical four-chamber showing biventricular hypertrophy, Large ASD (arrow), and color Doppler showing left to right shunt. C: 2D-Echocardiography parasternal short axis showing, dysplastic pulmonary valve (arrow), and severe pulmonary stenosis. D: 2D-Echocardiography showing LV gradient of 64.7 mm and peak pulmonary gradient of 149 mm. E: 2D and 3D-Echocardiography left parasternal long axis showing biventricular hypertrophy.

**Fig. 2:**
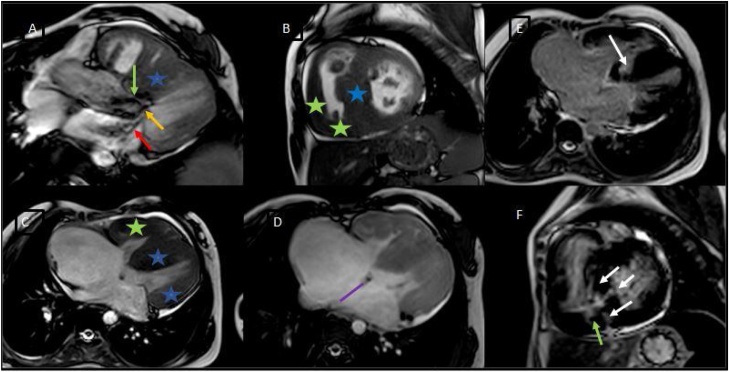
A: Still capture of the cine three-chamber view showing asymmetrical septal hypertrophy (blue star), the systolic anterior motion of the mitral valve (yellow arrow), left ventricular outflow tract flow acceleration (green arrow) and mitral regurgitation (red arrow). B: Still capture of cine short-axis view at the level of the mid ventricle in diastole showing asymmetrical septal hypertrophy (blue star) along with hypertrophy of the inferior and free of the wall of the RV (green stars). C: Still capture of the cine four-chamber view showing biventricular hypertrophy (green and blue stars). D: Still capture of the axial cine sequence showing the large ostiumsecundum atrial septal defect (purple line). E: Myocardial delayed enhancement sequence in the four-chamber view showing a mid-wall scar in the basal inferoseptal segment extending to the RV side of the septum. F: Myocardial delayed enhancement sequence in the short axis view showing mid-wall scar in the basal inferoseptal segment (white arrows) and the inferior RV wall (green arrow).

**Fig. 3:**
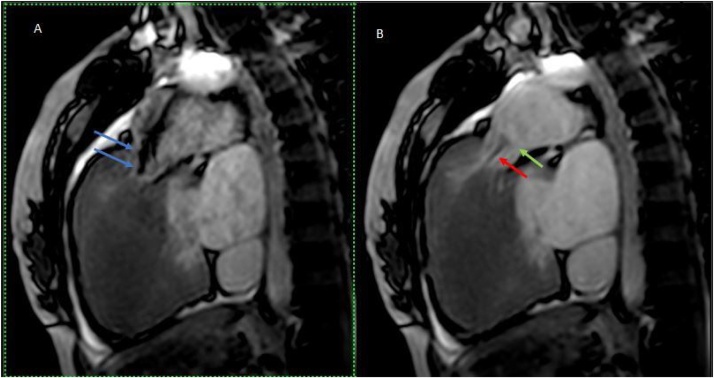
A: Still capture of cine sequence in systole of the right ventricular outflow tract showing sub-pulmonic/infundibular resting flow acceleration (blue arrows). B: Still capture of the cine sequence in diastole of the right ventricular outflow tract showing pulmonary regurgitation (red arrow). The level of the pulmonary annulus appreciated (green arrow).

**Fig. 4:**
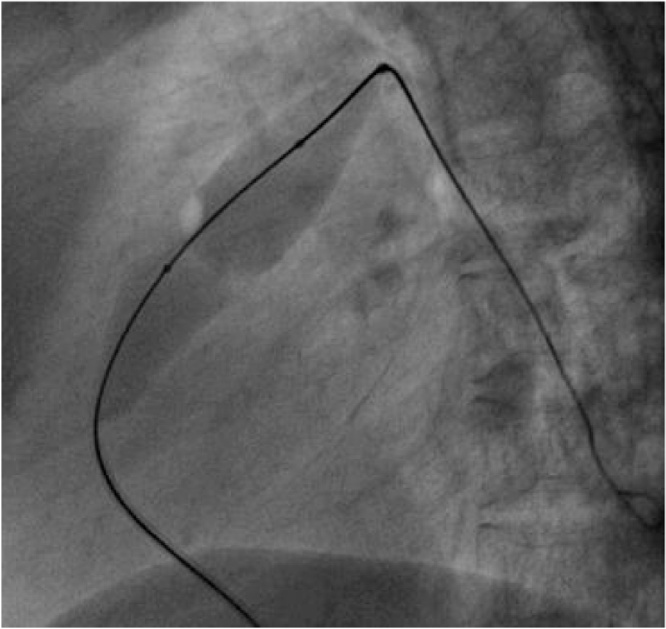
Pulmonary Balloon Valvuloplasty.

2D-Echocardiography showed biventricular hypertrophy, dysplastic pulmonary valve, severe pulmonary stenosis, and large ASD (Fig. 1A–D). 2D-Echocardiography and 3D-Echocardiography showed biventricular hypertrophy (Fig. 1E). CT Coronary Angiography revealed normal coronaries. MRI still capture of the cine three-chamber view showed asymmetrical septal hypertrophy, the systolic anterior motion of the mitral valve, left ventricular outflow tract flow acceleration, and mitral regurgitation (Fig. 2A). MRI still capture of cine short-axis view at the level of the mid ventricle in diastole showed asymmetrical septal hypertrophy, along with hypertrophy of the inferior and free wall of the Right ventricle (RV) (Fig. 2B). MRI still capture of the cine four-chamber view showed biventricular hypertrophy (Fig. 2C). MRI still capture of the axial cine sequence showed a large ostium secundum atrial septal defect (Fig. 2D). Myocardial delayed enhancement sequence in the four-chamber view showed a mid-wall scar in the basal inferoseptal segment extending to the RV side of the septum (Fig. 2E). Myocardial delayed enhancement sequence in the short axis view showed a mid-wall scar in the basal inferoseptal segment and the inferior RV wall (Fig. 2F). MRI still captures of cine sequence in systole of the right ventricular outflow tract showed sub-pulmonic/infundibular resting flow acceleration (Fig. 3A). MRI still captures of the cine sequence in diastole of the right ventricular outflow tract showed pulmonary regurgitation. The level of the pulmonary annulus can be appreciated in Fig. 3B. The genetic analysis report showed autosomal dominant inheritance with Ras/MAPK (mitogen-activated protein kinase) positive. Endomyocardial biopsy showed focal interstitial fibrosis and fiber hypertrophy and disarray. She underwent a percutaneous transluminal pulmonary valvuloplasty for pulmonary stenosis and was started on metoprolol succinate for hypertrophic cardiomyopathy (Fig. 4). The patient experienced significant symptomatic improvement after treatment, with 2D echocardiography showing decreased gradients across the pulmonary valve and left ventricular outflow tract. The cardiothoracic vascular surgical team planned for a heart transplant, and the patient is under regular follow-up.

## 3 Discussion

Noonan syndrome is one of the most common genetic disorders associated with congenital heart defects, being second only to Down syndrome [4]. It is a heterogeneous group of multiple congenital anomalies easy to recognize clinically. Noonan Syndrome is nearly always an autosomal dominant condition, and two-thirds of the patients are the first affected person in their family due to a de novo mutation [5]. As the child grows, the clinical diagnosis becomes more and more difficult since the changes are not easily noticed, and the risk of consequences of untreated anomalies such as cardiac anomalies, cryptorchidism, chest wall deformities, coagulation disorders, slow psychomotor development, strabismus and amblyopia increase during the growth [6]. Due to the broad spectrum of symptoms and presentations in Noonan cases, accurate clinical and genetic diagnosis, and comprehensive management of the disorder are strongly recommended. The Noonan Syndrome Support Group (NSSG) is a supportive organization involved in designing guidelines regarding the diagnosis and management of NS through recent genetic findings [7].

## 4 Conclusion

Patients with Noonan Syndrome have a distinct spectrum of cardiac phenotypes that may have a natural history and response to therapy atypical to that typically seen in non-syndromic heart disease. We have described a case of a rare combination of cardiovascular defects in Noonan Syndrome intending to achieve better insight into the disease course and the advantages of timely treatment and follow up. Our patient is currently in follow-up after treatment, with improved symptoms, and is awaiting Heart Transplant. With a deeper understanding of disease characteristics distinct to Noonan Syndrome, clinicians will be better able to counsel families regarding prognosis and tailor therapy for optimal patient outcomes.

## Declaration of Competing Interest

None.

## Funding

None

## Ethical approval

Committee Name: Sri Jayadeva Institute Ethics Committee.

Status: Approved.

## Consent

Written informed consent was obtained from the patient for publication of this case report and accompanying images. A copy of the written consent is available for review by the Editor-in-Chief of this journal on request.

## Author contribution

**Dr. H.S. Natraj Setty MD, DM, FICC, FRCP, FACC:** Study concept and design, data analysis, writing the paper.

**Dr. Shankar S MD, DM:** Data interpretation.

**Dr. Yeriswamy M.C MD, DM;** Data collection.

**Dr. Babu Reddy MD, DM:** Data interpretation.

**Dr. Jayashree Kharge MD, DM:** Data interpretation.

**Dr. T.R. Raghu MD, DM:** Data analysis.

**Dr. Sandeep Shankar MD, DM:** Data analysis.

**Dr. Rahul Patil MD, DM:** Data analysis.

**Dr. Sathwik Raj MD, DM:** Data interpretation.

**Dr. C.N. Manjunath MD, DM:** Final approval.

## Registration of research studies

1. Name of the registry: NA.
2. Unique identifying number or registration ID: NA.
3. Hyperlink to your specific registration (must be publicly accessible and will be checked): NA.

## Guarantor

Dr. H.S Natraj Setty.

## Provenance and peer review

Not commissioned, externally peer-reviewed.
